# A Diverse Group of Previously Unrecognized Human Rhinoviruses Are Common Causes of Respiratory Illnesses in Infants

**DOI:** 10.1371/journal.pone.0000966

**Published:** 2007-10-03

**Authors:** Wai-Ming Lee, Christin Kiesner, Tressa Pappas, Iris Lee, Kris Grindle, Tuomas Jartti, Bogdan Jakiela, Robert F. Lemanske, Peter A. Shult, James E. Gern

**Affiliations:** 1 Department of Pediatrics and Medicine, University of Wisconsin, Madison, Wisconsin, United States of America; 2 Wisconsin State Laboratory of Hygiene, University of Wisconsin, Madison, Wisconsin, United States of America; Medical University of South Carolina, United States of America

## Abstract

**Background:**

Human rhinoviruses (HRVs) are the most prevalent human pathogens, and consist of 101 serotypes that are classified into groups A and B according to sequence variations. HRV infections cause a wide spectrum of clinical outcomes ranging from asymptomatic infection to severe lower respiratory symptoms. Defining the role of specific strains in various HRV illnesses has been difficult because traditional serology, which requires viral culture and neutralization tests using 101 serotype-specific antisera, is insensitive and laborious.

**Methods and Findings:**

To directly type HRVs in nasal secretions of infants with frequent respiratory illnesses, we developed a sensitive molecular typing assay based on phylogenetic comparisons of a 260-bp variable sequence in the 5' noncoding region with homologous sequences of the 101 known serotypes. Nasal samples from 26 infants were first tested with a multiplex PCR assay for respiratory viruses, and HRV was the most common virus found (108 of 181 samples). Typing was completed for 101 samples and 103 HRVs were identified. Surprisingly, 54 (52.4%) HRVs did not match any of the known serotypes and had 12–35% nucleotide divergence from the nearest reference HRVs. Of these novel viruses, 9 strains (17 HRVs) segregated from HRVA, HRVB and human enterovirus into a distinct genetic group (“C”). None of these new strains could be cultured in traditional cell lines.

**Conclusions:**

By molecular analysis, over 50% of HRV detected in sick infants were previously unrecognized strains, including 9 strains that may represent a new HRV group. These findings indicate that the number of HRV strains is considerably larger than the 101 serotypes identified with traditional diagnostic techniques, and provide evidence of a new HRV group.

## Introduction

Human rhinoviruses (HRVs), members of picornavirus family, are small nonenveloped viruses with a 7200-base mRNA positive sense RNA genome [1]. The first HRV was discovered in 1956 [2], [3], and by 1987, 101 serotypes (1A and 1B to 100) were identified using susceptible cell cultures and specific antisera [4], [5], [6]. Multiple epidemiologic studies of serotype circulation conducted between 1975–1983 showed that >90% of field isolates could be identified with the 90 serotype-specific antisera prepared before 1973, and many serotypes identified earlier were still circulating [6], [7], [8]. These results suggested HRV serotypes are stable and do not undergo influenza virus-like antigenic drift [7].

HRVs are the most prevalent human respiratory pathogens [8], [9], [10], [11], [12]. Annually, HRVs are responsible for >50% of all acute upper respiratory illness (common colds), the most frequent human illness. HRV infections occur year round worldwide and are epidemic in early fall and late spring in the temperate regions. HRV infections cause a wide range of clinical outcomes including asymptomatic infections,[13], [14], [15], [16], [17] upper respiratory illnesses, and in children, asthmatics, and other susceptible populations, lower respiratory symptoms.[18], [19], [20], [21], [22], [23].

Defining the role of specific strains in various HRV illnesses has been difficult because traditional serology requires the isolation of HRV in susceptible cell cultures and neutralization tests against all 101 serotype-specific antisera [6]. This traditional serological method is insensitive, labor intensive and cumbersome [24]. More sensitive and faster molecular methods have been developed for serotyping enteroviruses, which are closely related to HRV [25]. In addition, molecular typing methods have been used to identify the links between illnesses and specific strains of pathogens such as dengue viruses, influenza viruses, human papillomaviruses, hepatitis C viruses, and HIV [26], [27]. Molecular typing involves PCR amplification of a portion of the target viral genome, sequencing and phylogenetic analyses. In this report, we analyzed clinical specimens from sick infants with a new molecular method, and identified 26 new HRV strains including 9 that constitute a new HRV group.

## Results

### Sequence variability of P1-P2 region between 101 HRV serotypes

**Figure 1:**
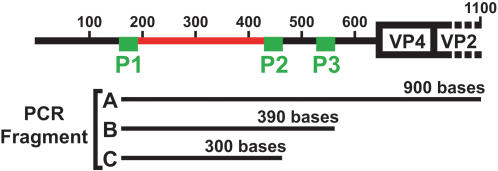
Schematic representation of the first 1100 base of a HRV genome showing the locations of the highly conserved regions (P1, P2 and P3) and variable region between P1 and P2 (P1-P2 in red) at the 5'NCR and the PCR fragments used in this study. P1, P2 and P3 are located at bases 163–181, 443–463 and 535–551, respectively in HRV16 genome. PCR fragment A (about 900bps) was used to determine the 5'NCR sequences of all 101 HRV serotypes. It was amplified using pan-HRV PCR forward primer P1-1, which anneals to conserved region P1, and a serotype-specific reverse primer annealed to the 5' end of VP2 gene (between base# 1000 and 1100). PCR fragment B (about 390 bps) was generated with pan-HRV PCR forward primer P1-1 and reverse primer P3-1. PCR fragment C (about 300 bps) was generated with forward primer P1-1 and an equimolar mixture of reverse primers P2-1, P2-2 and P2-3. The variable sequences of P1-P2 were used for the molecular typing assay.

**Figure 2:**
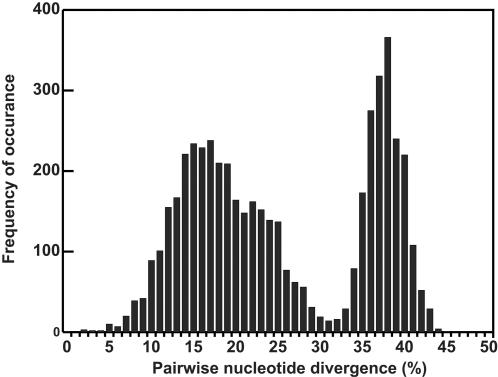
Distribution of pairwise nucleotide divergence values between 101 HRV serotypes. The horizontal axis shows the value of divergence (%) in pairwise comparisons and the column height indicates the frequency of observations. Divergence values were calculated as distance value×100%.

The length of the P1-P2 sequences (region between primer sites P1 and P2 in Figure 1) varied only slightly between the 101 established serotypes, ranging from 261 to 273 bases. The maximum pairwise nucleotide divergence (%) between all 101 serotypes in this region was 45% (Figure 2). This result was similar to the maximum pairwise divergence of 101 VP4 sequences (46%) and slightly lower than that of VP1 sequences (54%) [28], [29]. Furthermore, 97.5% of all the serotype pairs had >9% pairwise nucleotide divergence. The maximum pairwise divergences (%) of P1-P2 sequences among HRVA and HRVB viruses were 33% and 27%, respectively. These results demonstrated the potential utility of this region for differentiating HRV serotypes.

### P1-P2 sequences of 101 HRV serotypes clustered into 2 previously defined genetic groups: HRVA and HRVB

**Figure 3:**
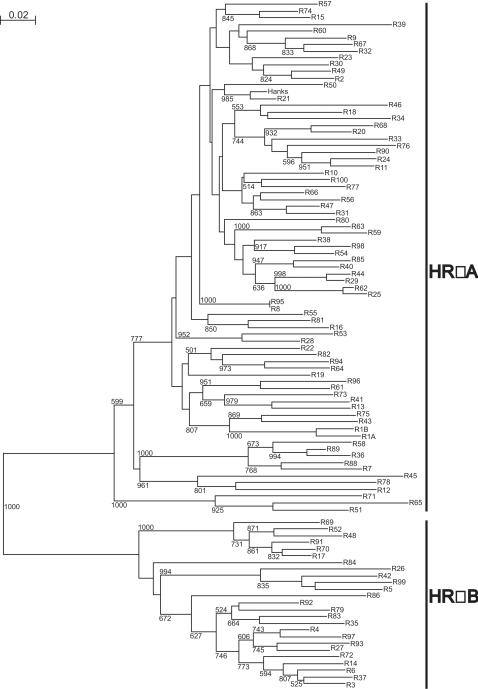
Phylogenetic tree depicting the relationships among all 101 HRV serotypes in the P1-P2 region of 5'NCR. This tree was constructed using the neighbor joining method according to the distances (divergences) between all pairs of sequences in a multiple alignment. The confidence of sequence clustering was evaluated by bootstrapping (1000 replicates). Only significant bootstrap values (>500) were shown. The scale bar (top left) represents the genetic distance (nucleotide substitutions per site). The 101 HRV serotypes (R) clustered into 2 previously defined groups: HRVA and HRVB with a perfect bootstrap value (1000). HRVA group had 76 serotypes and HRVB, 25 serotypes.

Phylogenetic tree reconstruction confirmed that the 101 P1-P2 sequences clustered into 2 genetic groups, A and B, (Figure 3). The P1-P2 phylogenetic distribution of the serotypes into group was identical to that of published trees based on VP1 and VP4-VP2 sequences [28], [29], [30], with the same 76 serotypes in the HRVA group and 25 serotypes in HRVB group (Figure 3). The topology of the P1-P2 tree was similar to that of the VP1 and VP4-VP2 trees [28], [29], [30]. These results agreed with previous reports that the nucleotide phylogenies of HRVs are consistent across the whole genome, from 5'NCR through polyprotein to 3'NCR [31].

### Accurate typing of 79 clinical isolates by phylogenetic tree reconstruction of P1-P2 sequences

To test whether P1-P2 sequences were suitable for HRV serotype identification, we compared the typing results of 79 culturable clinical isolates obtained by P1-P2 sequences with those by NIm-1A sequences of VP1. The degenerate primers EV292 and EV222 for PCR amplification of NIm-1A region were not sensitive enough for direct detection of small amount of HRV in original clinical samples (data not shown), and high titer infected cell lysates of cultured isolates were needed to produce enough PCR product for cloning and sequencing. The length of NIm-1A and P1-P2 sequences ranged 329–356 bases and 263–271 bases, respectively. Based on phylogenetic tree reconstruction of NIm-1A sequences, the 79 clinical isolates were assigned to 24 different serotypes with highly significant bootstrap values (77–100%, Table 1). Identical assignment results were obtained with P1-P2 tree, although a number of assignments (HRV1B, HRV15, HRV85 and one of the 5 HRV89) had low but still significant bootstrap values (60%, 64%, 61% and 62%, respectively). Interestingly, the nucleotide divergence between the clinical isolates and the respective reference strain was lower at the P1-P2 region (mean 3.5%, range 0–8%) than at the NIm-1A region (mean 9.6%, range 5–13%) (Table 1). This result agreed with previous reports that nucleotide sequences were more conserved at the NCRs than the coding region due to the preservation of conserved RNA structure elements within the NCR[31], [32].

**Table 1:** Identification of culturable HRV clinical isolates by phylogenetic tree reconstruction.

Serotype	# Isolates	NIm1A Region of VP1		P1-P2 Region of 5'NCR	
		Boot-strap Value (%)	Identity to Reference sequence (%)	Boot-strap Value (%)	Identity to Reference sequence (%)
HRV1B	1	86	90	60	97
HRV9	1	90	91	83	96
HRV11	1	100	91	99	98
HRV15	1	100	90	64	96
HRV18	1	100	94	90	96
HRV19	11	100	89–91	98–100	95–96
HRV21	2	100	92	93–98	97–98
HRV28	3	99–100	87–89	99–100	97
HRV34	3	95–100	90	100	95
HRV36	1	90	91	75	97
HRV38	7	100	90	98–100	92–98
HRV43	5	100	88	100	97
HRV47	5	99–100	90–91	87–91	97
HRV49	4	82–94	90	84	96–97
HRV54	4	93–95	90–91	85–95	98–99
HRV55	3	100	95	100	97
HRV56	9	96–99	88–89	81–94	93–95
HRV57	1	100	95	100	98
HRV60	1	100	90	99	95
HRV61	1	100	93	99	95
HRV65	1	100	94	100	99
HRV85	2	100	90–92	61	95
HRV89^a^	5	77–80	91–92	62–96	99–100
HRV98	6	80–98	91–95	95–100	97–98

a) Four isolates clustered with P1-P2 reference sequence with highly significant bootstrap values (96%), and one with lower but still significant bootstrap value (62%).

### HRV detection in clinical specimens

Nasal lavage samples of 181 illnesses from 26 infants with frequent respiratory illnesses were analyzed by Respiratory Multicode Assay [33]. HRV was detected in 108 samples (60%) (Table 2). Other viruses detected were enterovirus, RSV, adenovirus, coronavirus, influenza A virus, metapneumovirus and parainfluenza virus. Among the 108 HRV-positive samples, 80 had only HRV and 28 had coinfection with at least one other respiratory virus.

**Table 2:** Viruses detected in nasal samples of 26 infants with frequent respiratory illnesses by Respiratory Multicode Assay.

Viruses	No. of positive samples
HRV	108
Enterovirus	4
RSV A and B	24
Adenovirus	16
Coronavirus (OC43 and NL63)	15
Influenza A	14
Metapneumovirus	14
Parainfluenza	12
None	22
Multiple viruses^a^	41

a) 34 and 7 samples had 2 and 3 viruses, respectively.

The identity of HRV in 101 of the 108 samples was determined by molecular typing. Of the 7 samples that were not typed, 2 had no sample left and 5 did not yield P1-P2 fragments by semi-nested PCR. A total of 103 HRVs were identified in 101 samples. Only 2 samples contained 2 different HRVs, indicating a low rate of infection with more than one strain. Of the 103 HRVs, serotypes were assigned to 49 HRVs by phylogenetic tree reconstruction (Table 3). The assignments of 45 HRVs were strongly supported by highly significant bootstrap values (>74%). One assignment (HRV20) had a low but still significant bootstrap value (52%). The P1-P2 sequences of these 46 HRVs had 94–100% identity with the respective reference serotypes. Three sequences clustered with HRV89 but had poor bootstrap values (<50%) although they had 99% identity with HRV89 reference sequences. This was probably because these 3 HRVs also matched well with HRV36, which has 97% identity with HRV89 in the P1–P2 region. These 46 HRVs were grouped into 23 HRV serotypes; 42 (19 serotypes) were HRVA and 4 (4 serotypes) were HRVB viruses (Table 3).

**Table 3:** Detection of known HRV serotypes in clinical specimens.

Serotype^a^	# Detected	Boot-strap Value (%)	Identity to Reference Sequence (%)	Serotype^a^	# Detected	Boot-strap Value (%)	Identity to Reference Sequence (%)
HRV7	2	99–100	96–97	HRV63	1	96	98
HRV12	5	100	97–98	HRV78	6	100	95–99
HRV15	1	74	96	HRV80	1	100	98
HRV19	4	100	95–96	HRV82	1	100	98
HRV20	1	52	95	HRV89^b^	5	76–91	99–100
HRV28	3	100	97	HRV98	1	100	98
HRV33	2	100	94	HRV100	1	98	97
HRV38	1	93	95				
HRV47	1	78	97	HRV14	1	100	98
HRV49	3	85–92	97	HRV27	1	91	97
HRV54	1	96	99	HRV52	1	89	96
HRV61	2	93	95	HRV83	1	99	98

a) The first 19 serotypes are HRVA. HRV14, 27, 52 and 83, are HRVB.

b) Three additional HRVs clustered with HRV89 reference sequence with insignificant bootstrap values (42–50%).

### Identification of new HRV strains

**Figure 4:**
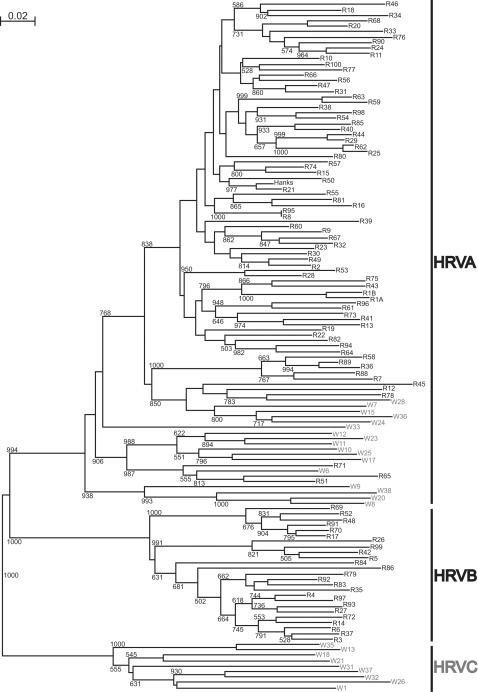
Phylogenetic tree depicting the relationships among 101 HRV serotypes (R) and 26 new strains (W) in the P1-P2 region of 5'NCR. This tree was generated as described in Figure 3. None of the new strains clustered with HRVB viruses. Seventeen new strains (blue) belonged to HRVA group, and 9 strains (red) cluster into a new group (“C”) that is separate from groups A and B.

The P1-P2 sequences of 54 HRVs did not cluster with the homologous sequences of any of the 101 serotypes in phylogenetic tree reconstructions (Figure 4). These 54 HRVs clustered into 26 new unique strains with high degree of nucleotide divergence from the respective nearest reference serotypes (mean 24.6%, range 12–35%) and between strains (mean 32.5%, range 10–46%) (Table 4). Different new HRVs of the same strain had a high degree of identity among themselves (mean 98.4%, range 94–100%, Table 4). Seventeen of the new strains clustered with group A HRV (Figure 4) and had 12–35% pairwise nucleotide divergence from the nearest reference serotype (Table 4). Among them, 5 strains (W7, W15, W24, W28 and W36) clustered in a distant branch near serotypes 12, 45 and 78; 7 strains (W6, W10, W11, W12, W17, W23 and W25) in distant branch near serotypes 51, 65 and 71; strains W8, W9, W20 and W38 formed a new branch, and strain W33 was the lone member of a new branch. Moreover, 9 strains (W1, W13, W18, W21, W26, W31, W32, W35 and W37) separated from both HRVA and HRVB (Figure 4) and had 31–35% pairwise divergence from the nearest reference serotype (Table 4). We propose that they represent a new HRV genetic group (HRVC). None of the new strains clustered with HRVB viruses.

**Table 4:** Detection of novel HRV strains in clinical specimens.

New HRV Strain	# Detected	Divergence from the Nearest Refererence. Sequence (%)	Identity Among HRVs of the Same Strain (%)	New HRV Strain	# Detected	Divergence from the Nearest Refererence. Sequence (%)	Identity Among HRVs of the Same Strain (%)
W1	5	32–33	97–100	W21	1	33	
W6	3	15	95–100	W23	4	20–21	99–100
W7	2	18–20	97	W24	3	18–19	95–99
W8	1	27		W25	1	23	
W9	2	23–24	100	W26	3	34–35	97–100
W10	2	22	98	W28	4	12–13	99–100
W11	1	17		W31	1	35	
W12	3	18	97–100	W32	2	31	99
W13	1	31		W33	1	24	
W15	1	13		W35	1	33	
W17	1	21		W36	1	18	
W18	2	32	100	W37	1	35	
W20	3	25–26	98–100	W38	3	35	94–99

Interestingly, none of the samples containing the new HRV strains produced CPE in standard WI-38 or MRC-5 cell cultures used for the detection and isolation of HRV (data not shown).

### HRVC was distinct from human enterovirus (HEV)

**Figure 5:**
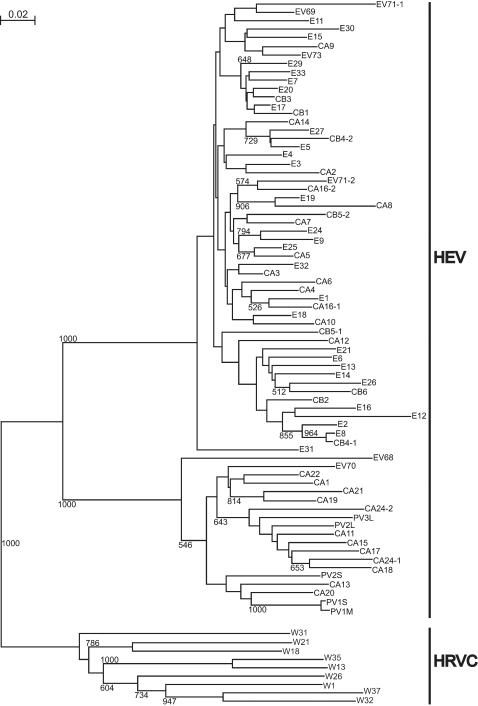
Phylogenetic tree depicting the relationships between 9 HRVC strains (W in red) and all known HEV (n = 74) in the P1-P2 region of 5'NCR. HEV include polioviruses (PV), coxsackieviruses A (CA), coxsackieviruses B (CB), echoviruses (E) and the newer numbered EVs. For 5 serotypes (CA16, CA24, CB4, CB5 and EV71), 2 different P1-P2 sequences were found in GenBank. This tree was generated as described in Figure 3. HRVC and HEV clustered into 2 different groups.

HEVs are closely related to HRVs [1], and comprise >65 distinct serotypes that include polioviruses, coxsackieviruses A and B, echoviruses and the newer numbered EVs. HEVs are classified into 5 groups: poliovirus and human enterovirus A-D (HEV-A-D), according to both biological and molecular properties. Like HRVs, some HEVs are upper respiratory pathogens. To determine the relationship of HRVC to HEVs, phylogenetic tree reconstruction was performed with the P1-P2 sequences of 9 HRVC strains and respective sequences of all known HEV (n = 74). The results indicate that HRVC viruses are distinct from HEV: the pairwise divergence of P1-P2 sequences between a HRVC strain and the respective nearest HEV ranged from 31% to 35% (Figure 5).

## Discussion

In this report we demonstrate that the pool of circulating HRV strains is significantly larger than the collection of 101 known serotypes. Less than half of the HRV detected in these young infants corresponded to previously recognized serotypes (Table 3), and 54 are previously unrecognized HRVs belonging to 26 new strains (Table 4). Moreover, 9 of the strains form a new genetic group "C" that is distinct from the previously defined groups HRVA and HRVB and the human enteroviruses (Figure 5). The remaining 17 new strains cluster into existing distant or new branches in HRVA group (Figure 4). Interestingly, none of the new viruses grew in standard tissue culture, which may explain why these viruses were previously undetected.

These new HRV strains were detected with a sensitive molecular method to type HRV directly from the original clinical specimens. This new assay had 3 key components: sensitive pan-HRV primers and semi-nested PCR to amplify P1-P2 region from cDNA prepared from original clinical specimens, a sequence database of 260-bp P1-P2 region of 5'NCR of all 101 HRV serotypes to serve as standard references for HRV identification, and phylogenetic tree reconstruction of the new P1-P2 sequences and the 101 homologous reference sequences. Phylogenetic tree reconstruction has been shown to be more accurate than the other sequence analysis methods for molecular typing of enteroviruses [34]. Interestingly, the 5'NCR is not suitable for typing of enterovirus. For example, the 5'NCR sequence of coxackieviruses do not correlate with serotype and VP1 sequence due to frequent recombination at the 5’NCR [35], [36]. In contrast, HRV maintains consistent phylogeny across its genome and has limited recombination [31], and this characteristic enables 5'NCR sequence (P1-P2) to be used for strain identification. For enteroviruses, VP1 sequencing is commonly used for molecular serotyping because this region contains major antigenic sites that correlate well with serotype [25]. However, VP1 sequences of HRV are less conserved, and degenerate primer pairs such as EV292 and EV222 [24] that target this region were relatively insensitive for PCR amplification.

The 101 established HRV serotypes (HRV1A to HRV100) were discovered and designated between 1956 and 1987. They were isolated using susceptible WI-38 cell cultures and then defined with serotype-specific antisera [4], [5], [6]. Multiple epidemiologic studies of serotype circulation conducted between 1975–1983 showed that >90% of the field isolates could be identified with the 90 serotype-specific antisera (HRV1A through HRV89) prepared before 1973 and many serotypes identified earlier were still circulating [8], [11] [6], [7]. These results suggested that almost all HRV serotypes had already been identified and new serotypes were not evolving [6], [7]. In fact, only one possible new serotype, HRV-Hanks, was reported in the next 20 years (1987 to 2006), and it was subsequently shown to be HRV21 by careful sequence analysis and neutralization testing [30].

Recently, studies utilizing molecular techniques instead of culture-based diagnostics have provided evidence of additional strains of HRV [37], [38]. For example, Lamson and colleagues identified 8 new HRVs in New York State (NY) by VP4 sequencing. They concluded that these HRVs represented a new genetic clade because they clustered in a branch at the root of the HRVA phylogenetic tree [37]. In addition, McErlean and colleagues obtained the complete genome sequence of a new HRV strain, HRV-QPM, in Queensland, Australia. They showed by phylogenetic analysis that HRV-QPM was a new member of HRVA and belonged to a new genetic sub-lineage of HRVA, HRV-A2, and the 8 new NY HRVs also belonged to HRV-A2 [38].

To compare the identities of these newly reported HRVs with our new strains, we performed phylogenetic tree reconstruction using P1-P2 sequences of our new strains, HRV-QPM, and the 101 established serotypes; and then a similar analysis using VP4 sequences of HRV-QPM, 8 NY HRVs, and the 101 established serotypes. The P1-P2 phylogenetic tree (not shown) revealed that HRV-QPM and one of our new HRVA viruses, W24, were the same strain and thus supported McErlean's conclusion that HRV-QPM was a group A virus. The VP4 tree (not shown) confirmed McErlean's finding that HRV-QPM and the 8 NY HRVs belonged to the same cluster within HRVA group. Therefore the 8 NY HRVs and HRV-QPM were related to our 17 new HRVA strains. In contrast, our 9 HRVC strains form a distinct group separated from both HRVA and HRVB (Figure 4).

Analysis of 5'NCR of all HRV serotypes reveals that there are both highly conserved sequences (e.g. P1, P2 and P3 primer sites, Figure 1) and also variable sequences between P1 and P2. The 260-bp P1-P2 region had up to 45% pairwise nucleotide divergence between serotypes, similar to that of VP4 (46%) and VP1 (54%). Moreover, 97.5% of all the P1-P2 pairs from distinct serotypes had >9% pairwise nucleotide divergence. Despite this inter-serotype variability, the P1-P2 sequence of 5'NCR was significantly more conserved between clinical isolates and the corresponding reference prototype strain (mean divergence 3.5%) compared to the NIm-1A coding sequences of VP1 (mean divergence 9.6%, Table 1). These data suggest that 5'NCR sequences may be under greater selective constraint, and it is known that this region contains RNA structure elements that are critical for viral replication and translation [32].

In summary, more than half (52%) of the HRVs detected in these young infants were new HRV strains, and many of them clustered into a distinct genetic group C. These findings indicate that the number of HRV strains has been markedly underestimated by traditional viral culture and serotyping techniques, and also raise additional questions. First, how widespread are these putative “group C” HRV? Our study population consisted of a group of infants who experienced frequent illnesses, and additional studies are needed to define the spectrum of HRV infections in unselected populations, and in subjects of other age groups. Secondly, is the biology of these novel strains similar to that of other HRV? The inability to culture these viruses suggests that receptor utilization or other growth requirements are distinct. The development of molecular assays for the detection and analysis of respiratory viruses provide important tools for new epidemiologic and mechanistic studies to address these questions. A more complete understanding of the spectrum of respiratory viruses and their biology is essential for efforts directed at prevention and treatment of these common and clinically significant illnesses.

## Materials and Methods

This study was approved by the Human Subject Committee of the School of Medicine and Public Health, University of Wisconsin -Madison. Written informed consent was obtained from the parents.

### HRV samples and clinical specimens

For determining the standard reference sequences, infected cell lysates of prototype strains of 101 HRV serotypes were obtained from Dr. Fred Hayden of U. Virginia, Charlottesville (serotype 1A, 1B, 2 to 10, 12 to 89, 91 to100 and Hanks) and ATCC (HRV11 and HRV90). HRV87 was excluded from the reference database because it has been reclassified as a human enterovirus [39], [40].

Clinical samples were obtained from two sources. First, samples were obtained from infants ages 0–1 year participating in a prospective birth cohort study (Childhood Origins of Asthma) in Wisconsin to determine the role of viral and host factors in the pathogenesis of asthma [41]. Of the 285 children who completed the first year of the study, 27 infants had frequent (≥5) moderate to severe respiratory illnesses, and samples from 26 were available for further study. Nasal lavage specimens were collected from 181 illnesses between spring of 1999 and spring of 2001. Second, for validation of our molecular typing assay, infected cell lysates of 79 additional HRV clinical isolates were obtained from Wisconsin State Laboratory of Hygiene (WSLH). These clinical isolates were recovered from nasal lavages of infants in 1999 and 2000 using WI38 cell culture and identified by the characteristic cytopathic effect and acid lability of HRV [42].

### Sequence analysis

Pairwise sequence alignment, multiple sequence alignment, % identity calculation, distance (divergence) calculation, phylogenetic tree reconstruction and bootstrap analysis were performed using software Clustal×1.8.3 [43], [44]. In a typical analysis, the input sequences were first processed to produce a multiple alignment and matrixes of identity and distances (divergence) between all sequence pairs. The distance matrix was used by the neighbor joining method to produce an unrooted phylogenetic tree with branch length proportional to the divergence of the sequences. The confidence of the clustering of sequences was evaluated by bootstrapping (1000 replicates). Bootstrap values of >700 (70%) indicate the highly significant clustering, whereas values <500 (50%) indicate that the clustering is not statistically significant. The phylogenetic tree with bootstrap values was visualized using software NJplot.

### Preparation of cDNA from nasal specimens

cDNA preparation was performed as described elsewhere [33]. Briefly, nasal fluid (350 µl) was mixed with extraction carriers (glycogen and glycoblue) and 750 µl of Trizol LS (Invitrogen 10296), vortexed for 10 minutes, supplied with 230 µl of chloroform, vortexed again for 5 minutes and then microfuged for 5 minutes. The supernatant aqueous phase (∼700 µl) was mixed with 600 µl isopropanol and this mixture was incubated at room temperature for 1 hr. The RNA precipitant was pelleted by microfugation for 10 minutes, washed once with 75% ethanol, air-dried and then dissolved in 20 µl water. To make cDNA, 16 µl of RNA solution was mixed with 24 µl of reaction solution containing Promega AMV-reverse transcriptase, AMV-RT buffer, random primers, RNAsin and dNTPs and then incubated at 25°C for 5 minutes, 42°C for 10 minutes, 50°C for 20 minutes, and 85°C for 5 minutes.

### Identification of HRV and other respiratory viruses in clinical specimens by Respiratory Multicode Assay (RMA)

RMA is a new high-throughput, multiplex PCR-microsphere flow cytometry assay system for comprehensive detection of common respiratory viruses including rhinoviruses (HRV), enteroviruses, respiratory syncytial viruses (RSV), parainfluenza viruses, influenza viruses, metapneumoviruses, adenoviruses and coronaviruses. Details of the RMA assay have been previously described [33]. The output signal is expressed as MFI (median fluorescence intensity), and samples with an average signal >6 standard deviations of average negative control signals (typically 400 to 500 MFI) are regarded as positive. The RMA is capable of distinguishing closely related HRV and enteroviruses [33].

### Development and validation of the molecular typing assay

#### Selection of the target region

To identify a genomic region suitable for molecular typing of HRV, we analyzed all published HRV sequences. These included complete genome sequences of 8 serotypes (1B, 2, 9, 14, 16,39, 85 and 89) [45], [46], [47], [48], [49], [50], [51], [52] and the Picornavirus Home Page [http://www.iah.bbsrc.ac.uk/virus/Picornaviridae/],VP1 and VP4-VP2 sequences of all serotypes [28], [29], [30], 3D (RNA polymerase) sequences of 48 serotypes [53] and partial 5'NCR sequences of 37 serotypes [54], [55], [56]. Careful alignment analysis of these sequences showed that only the 5'NCR region had highly conserved sequences (P1, P2 and P3 regions, Figure 1) that could be used for making pan-HRV primers, and a long variable sequence (P1-P2, Figure 1) suitable for serotype differentiation. However, 5'NCR sequences were available for only a fraction of 101 serotypes.

#### Cloning and sequencing of the 5'NCR of 101 reference HRV serotypes

To establish a reference sequence database for molecular typing, we cloned and sequenced the 5'NCR of all 101 HRV serotypes. Detailed viral RNA preparation, RT-PCR, cloning and sequencing procedure have been described elsewhere [33]. Briefly, total nucleic acids were prepared from 100 µl of infected cell lysate by phenol extraction and ethanol precipitation. RT (reverse transcription)-PCR was performed in a RT-PCR mix (Invitrogen 11922-028) using the following conditions: 30 min at 50°C, 2 min at 94°C, 33 cycles of (30 sec at 94°C, 30 sec at 50°C, 60 sec at 68°C) and 5 min at 68°C. The PCR primer pairs were forward primer P1-1 (CAAGCACTTCTGTYWCCCC) for all serotypes and a serotype-specific reverse primer. Primer P1-1 was designed within the conserved P1 region (Figure 1). The reverse primer was selected for each of the 101 serotypes within a region corresponding to bases 1000-1100 of HRV16 near the 5' end of VP2 gene (Figure 1) according to published sequences [29]. The PCR product, a DNA fragment of about 900 bp covering 75% of the 5'NCR, complete VP4 gene and about 200 bases of VP2 gene (PCR fragment A of Figure 1), was isolated by agarose gel electrophoresis. After the agarose was removed by phenol extraction, PCR fragments were treated with kinase, ligated to a StuI-linearized plamsid vector pMJ3, and then transformed into E. coli. Three plasmids with PCR fragment inserted were isolated for each serotype, amplified and purified. Each viral DNA fragment was completely sequenced (Automated DNA Sequencing Facility, U. Wisconsin). The serotype identity of each sequence was verified by matching of its VP4/VP2 sequence to the respective published sequence [29].

#### Semi-nested PCR amplification of P1-P2 region from original clinical specimens

Primer P1-1, which amplified the 5'NCR of all 101 serotypes, was chosen as the forward primer. For reverse primers, multiple candidates were designed within highly conserved P2 and P3 regions (Figure 1). Primers P3-1 (ACGGACACCCAAAGTAG), P2-1 (TTAGCCACATTCAGGGGC), P2-2 (TTAGCCACATTCAGGAGCC) and P2-3 (TTAGCCGCATTCAGGGG) were selected based on efficient HRV sequence amplification.

For the first PCR, 2.5 µl of leftover cDNA from the RMA assay was added to a tube containing 23 µl Platinum PCR SuperMix HF (Invitrogen 12532-016), 1 µl of forward primer P1-1 (25 µM) and 1 µl of reverse primer P3-1 (25 µM). The reaction started with 2 min at 94°C, followed by a 'touchdown' cycle (The steps were 94°C for 20 s, 68°C down to 52°C (2°C intervals) for 30 s, and then 68°C for 40 s. There were two cycles for each annealing temperature down to 54°C followed by 12 cycles at 52°C, and then a final 5 min at 68°C. This reaction produces a PCR product of 390 bp (Figure 1, fragment B). For the second PCR, 5 µl of the first PCR product was transferred to a new PCR tube containing 50 µl Platinum PCR SuperMix HF, 1 µl of forward primer P1-1 (25 µM) and 1 µl of each reverse primer P2-1 (25 µM), P2-2 (25 µM) and P2-3 (25 µM). The reaction conditions are 2 min at 94°C, 28 cycles of (20 sec at 94°C, 30 sec at 52°C, 40 sec at 68°C) and 3 min at 68°C. The final product is a 300 bp DNA fragment (Figure 1, fragment C). This semi-nested PCR protocol requires only 10 copies of cDNA template per sample to produce sufficient product for cloning and does not produce nonspecific product from original clinical specimens (data not shown). Fragment C was then purified, cloned, and 3 plasmids containing fragment C were isolated and sequenced for each sample. For 2 samples, 2 different P1-P2 sequences were found, indicating the presence of more than one serotype/strain, so 6 additional plasmids were analyzed.

#### Sequencing of the VP1 and 5'NCR of 79 HRV clinical isolates

To determine whether the P1-P2 variable sequences within the 5'NCR were suitable for the differentiation and identification of HRV, we compared the typing results by P1-P2 sequences with those of NIm-1A sequences of VP1. NIm-1A is a dominant antigenic site of HRV first identified for HRV14 [1], and the homologous sequences of this region correlate well with serotype of HRVs and enteroviruses [57], [58]. Viral RNA was isolated from virus-infected culture supernatant of 79 HRV clinical isolates with QIAamp viral RNA mini kit (Qiagen 52904) as described [24]. To obtain the VP1 sequences, viral RNA was first amplified using primer pair EV292 and EV222 as described [24] with modified RT-PCR conditions: 10 min at 38°C, 40 min at 50°C, 3 min at 95°C, 40 cycles of (30 sec at 95°C, 45 sec at 42°C, 30 sec at 65°C) and 1 min at 70°C. The PCR product (∼350 bp) was isolated by agarose gel electrophoresis and then sequenced [24]. To obtain the 5'NCR sequences, viral RNA was amplified by RT-PCR using P1-1 and P3-1 primers, cloned and sequenced.

#### Assignment of serotypes and new strain

A phylogenetic tree with bootstrap values and a matrix of % pairwise nucleotide divergence (distance×100%) were generated for each new sequence (NIm-1A or P1-P2) and 101 homologous reference sequences using Clustal×with default alignment parameters, which were selected after testing a range of parameters. Next, a new isolate or detection was assigned the serotype to which it clustered with in the phylogenetic tree with a significant bootstrap value (>50%) [34]. In contrast, if the P1-P2 sequence of a new isolate or detection did not cluster with one of the 101 serotypes in the phylogenetic tree, and had >9% pairwise nucleotide divergence from the nearest reference serotype, it was designated as a new strain (prefixed with W). The threshold pairwise divergence value (9%) for assigning a new strain was determined after considering the pairwise divergence values between the 101 known serotypes (Figure 2), and the pairwise divergence values between reference serotypes and clinical isolates of the same serotype (Table 1, last column).

Two types of errors could be made in evaluating two sequences: A) deciding they were the same when they were actually different, and B) deciding they were different when they were actually the same. The probability of these errors was determined empirically using the data shown in Figure 2 and Table 1, respectively. According to Figure 2, 99%, 98%, 97%, 95%, and 90% of the P1-P2 pairs of all 101 serotypes had >7%, >8%, >9%, >10% and >12% pairwise nucleotide divergence, respectively. As shown in Table 1 (last column), the pairwise nucleotide divergence of all P1-P2 pairs between clinical isolates and the respective reference serotypes was consistently ≤8%. Therefore, a threshold of 9% is associated with probabilities of <3% and <1% for errors A and B, respectively.

### Nucelotide sequence accession numbers

The original P1-P2 sequences described in this report have been deposited in the GenBank sequence database under accession no. EU126663 to EU126789.
